# Unveiling the Cytotoxicity Potential of Nanoemulsion of *Peltophorum pterocarpum* Extract: A Natural Hemocompatible Injection Competing with Doxorubicin

**DOI:** 10.3390/ph18121818

**Published:** 2025-11-28

**Authors:** Al Zahraa G. Al Ashmawy, Afaf E. AbdelGhani, Wafaa H. B. Hassan, Fatma O. El Weshahy, Wael M. Abdelmageed, Shaza M. Al-Massarani, Omer A. Basudan, Aalaa Gamil, May Ahmed El-Sayed

**Affiliations:** 1 Department of Pharmaceutics and Pharmaceutical Technology, Faculty of Pharmacy, Obour University for Science and Technology, Cairo 11828, Egypt; 2 Department of Pharmacognosy, Faculty of Pharmacy, Zagazig University, Zagazig 44519, Egypt; aeabdelghani@zu.edu.eg (A.E.A.); wafaahbh@zu.edu.eg (W.H.B.H.); fosoliman@pharmacy.zu.edu.eg (F.O.E.W.); mayalsayed@zu.edu.eg (M.A.E.-S.); 3 Department of Pharmacognosy, Faculty of Pharmacy, Assiut University, Assiut 71526, Egypt; wabdelmageed@aun.edu.eg; 4 Department of Pharmacognosy, College of Pharmacy, King Saud University, P.O. Box 2457, Riyadh 11451, Saudi Arabia; salmassarani@ksu.edu.sa; 5 Department of Clinical Oncology, Helwan University, Helwan 11795, Egypt; aalaagamil100@gmail.com

**Keywords:** bergenin, bioavailability, transmission electron microscopy, hemocompatibility, hepatocellular carcinoma

## Abstract

**Background/Objectives**: According to the WHO, more than one million deaths of liver cancer patients will occur in 2030. Hepatocellular carcinoma (HCC) is the third leading cause of death among all cancer types. Doxorubicin is commonly used for the treatment of HCC, yet it possesses major side effects. The aim of this work was to formulate a nanoemulsion of *Peltophorum pterocarpum* extract containing bergenin intended for intravenous injection as a natural alternative to doxorubicin. **Methods**: The saturation solubility of the extract in different oils, surfactants, and co-surfactants was determined. Surfactant to co-surfactant mixtures (Smix) were used at six different weight ratios. A pseudoternary phase diagram was constructed, and the ratio with the highest area was chosen. Six formulations were prepared by changing the oil-to-Smix ratio. They were evaluated by percentage transmission, dilution test, self-emulsification, pH, viscosity, drug content, droplet size, PDI, zeta potential, TEM, in vitro drug release, stability, in vitro hemolysis percentage, and cytotoxicity (for the optimized formula). **Results**: F6 of oil-to-Smix ratio (1:6) was chosen for further investigations, as it possesses the lowest droplet size, the highest zeta potential, drug content, and in vitro drug release. The pH, viscosity, and self-emulsification time of F6 were also acceptable. F6 possesses shelf-life stability and is hemocompatible. It possesses high cytotoxicity against the HepG-2 cell line (IC_50_ = 14.19 µg/mL). **Conclusions**: Although the nanoemulsion is less potent than doxorubicin in terms of IC_50_, it offers a safer profile and natural origin, which may be used for the treatment of HCC.

## 1. Introduction

The genus *Peltophorum* belongs to the family Fabaceae and includes approximately 105 species [1]. *Peltophorum pterocarpum* (*P. pterocarpum*) is a member of this genus and is commonly known as golden flamboyant, yellow poinciana. It is native to the Indo-Malaysian region. *P. pterocarpum* is a fast-growing deciduous tree that typically reaches a height of about 15 m [2]. Different parts of *P. pterocarpum* are traditionally used to treat various diseases such as stomatitis, insomnia, skin disorders, constipation, ringworm, and malaria. In Southeast Nigeria, the stem bark is commonly used in the management of malaria and bacterial infections. Previous studies have shown that the pods of *P. pterocarpum* possess antioxidant and anti-hemolytic activities [3,4,5]. Additionally, the aqueous extract of its flowers has demonstrated significant antibacterial and antifungal properties [6]. It possesses a cholinesterase inhibitory effect, suggesting its potential use in managing neurodegenerative disorders such as Alzheimer’s disease [7].

A literature survey on the *P. pterocarpum* plant reveals the presence of sterols and/or terpenes, flavonoids, coumarins, alkaloids, amino acids, vitamin E, aliphatic alcohol, and tannins in addition to acids [7]. Bergenin is an isocoumarin derivative in *P. pterocarpum* [7]. In China, bergenin is widely used as a famous antitussive for the treatment of chronic bacterial infection of the trachea (tracheitis) [8]. Bergenin possesses many pharmacological actions, including anti-inflammatory, antiviral, antibacterial, antifungal, antimalarial, anti-obesity, hepatoprotective, and anticancer effects [9,10]. Bergenin has demonstrated anticancer activity in various models, such as hepatocellular carcinoma, cervical cancer, bladder cancer, prostate cancer, colorectal cancer, and others [10]. The anticancer effect of bergenin emerged as a result of being an antioxidant, causing apoptosis and angiogenesis, and decreasing signaling pathways of cancer cells [10]. Despite being a very effective treatment for many diseases, bergenin is unfortunately classified as a class IV chemical possessing low solubility and permeability in oral absorption [10].

Hepatocellular carcinoma (HCC) is a major health problem [11,12]. Worldwide, HCC ranks as the 6th most common cancer and the 3rd most common cause of cancer-related mortality [12,13,14]. In 2020, 906,000 new cases were diagnosed as HCC patients, and 830,000 deaths occurred globally [15]. In Egypt, HCC has the highest incidence and mortality rates among malignant tumors [16]. Over the next 30 years, there will likely be a significant increase in HCC cases [13]. Recent data also emphasize the role of genetic mutations in HBV-associated HCC progression, such as rtA181T, which correlates with elevated risks of carcinogenesis due to increased mutation rates in tumor suppressor genes [17]. Various treatment modalities for HCC are available, including resection, liver transplantation, local ablation by radiofrequency (RFA) or microwave (MWA), arterial-directed therapies such as trans-arterial chemoembolization (TACE), trans-arterial radioembolization (TARE), and systemic treatment [18]. Systemic treatment for advanced HCC not amenable to local therapies has many advances that started from oral tyrosine kinase inhibitors, such as sorafenib, lenvatinib, regorafenib, and cabozantinib, ending with immunotherapy, either combination with anti-vascular endothelial growth factor receptors (VEGF) with atezolizumab, bevacizumab, immuno-immuno combination as durvalumab tremelimumab or single-agent immunotherapy as nivolumab or pembrolizumab [19]. Management of HCC depends on tumor stage, underlying liver condition, and patient’s performance status and requires a multidisciplinary team approach [18].

According to HCC management guidelines of the European Association for the Study of the Liver (EASL) [20] and the American Association for the Study of Liver Diseases (AASLD) [21], trans-arterial therapies are recommended in the treatment of multinodular HCC. Trans-arterial chemoembolization is an effective treatment leading to median survival rates [22]. The idea of TACE is based on blocking the blood supply of the tumor by selective injection of chemotherapy mixed with lipidol into the hepatic artery, leading to tumor necrosis due to both the cytotoxic effect of chemotherapy and ischemia of tumor cells [23]. Many chemotherapeutics were tested in TACE either as a single agent or in combination; however, the most widely used is doxorubicin, 35 to 75 mg/m^2^ emulsified in 5 to 20 mL of lipidol [24]. Unfortunately, doxorubicin has side effects such as nausea, vomiting, stomatitis, blood count suppression, and alopecia [25]. Among its chronic toxicities are dilated cardiomyopathy and congestive heart failure [25]. Among local side effects accompanied by doxorubicin use is skin necrosis as a result of extravasation and radiation [25]. To overcome these limitations, we developed a nanoemulsion of *P. pterocarpum* extract, which combines the anticancer potential of bergenin with the safety and delivery advantages of nanotechnology. Recent advances in liver injury models have revealed the potential of natural products to modulate the gut-liver axis and inflammatory responses, as shown by Guo et al. (2025), where Zingibroside R1 from Achyranthes bidentata mitigated chemically induced hepatic injury via gut microbiota regulation [26].

Over many years, nanoemulsion has proved its efficacy as a parenteral dosage form, dissolving both hydrophilic and hydrophobic drugs [27]. Nanoemulsion offers many advantages, as it increases drug solubility, absorption, bioavailability, and stability. Additionally, recent breakthroughs in precision engineering of nanocarriers and prodrug strategies have demonstrated significant improvements in doxorubicin’s therapeutic index, which underlines the importance of smart nanoformulations in modern oncology [28]. Nanoemulsion extends the drug’s shelf-life, preventing its deterioration [29]. Nanoemulsion is safe and cost-effective [27,29]. Nanoemulsion also has a high ability to load drugs so that it can deliver different therapeutic agents. Going back to ancient civilizations, herbal medicines were widely used for the treatment of many diseases [30]. Approximately 80% of the world’s population relies on herbal medicines for primary health care [31]. Herbal medicines may possess low solubility, bioavailability, and stability [32]. Nanotechnology was widely used in herbal medicines for increasing their efficacy and bioavailability and decreasing their adverse effects [32,33]. The use of nanotechnology in herbal medicines extends their uses for the treatment of many chronic diseases, such as diabetes, cancer, and cardiovascular diseases [32]. In this study, *P. pterocarpum* extract was incorporated into a nanoemulsion intended to be intravenously injected for proper treatment of HCC as an alternative treatment to doxorubicin to avoid its major side effects.

## 2. Results and Discussion

### 2.1. Standardization of Bergenin in P. pterocarpum Extract Using HPLC

The standardization step is very important to determine the concentration of bergenin in *P. pterocarpum* extract. Bergenin is a C-glycoside of 4-O-methyl gallic acid [10]. Bergenin is a major compound in *P. pterocarpum* extract [34]; it may be responsible for the pharmacological actions and anticancer effect [10], so quantification of bergenin concentration in the extract was a must to determine the correct dose. Figure S2, Supplementary Materials, shows the HPLC chromatogram of pure bergenin at 275 nm; the retention time of bergenin was 11 min. The regression equation was Y = 28,182x − 7518.1. x is the concentration of the standard samples (µg/mL), and the correlation coefficient R^2^ was 1. The percentage of bergenin in the leaf of *P. pterocarpum* extract was 2%. This result is in agreement with Muniz et al. (2020), who reported that the bergenin methanolic extract of Endopleura uchi contains 2.23% of pure bergenin [35].

### 2.2. Saturation Solubility of P. pterocarpum Extract Using Different Oils, Surfactants, and Co-Surfactants

**Figure 1:**
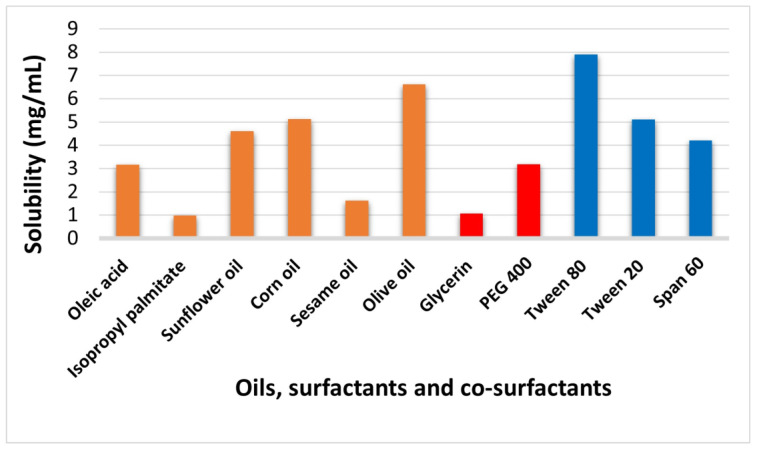
Saturation solubility of *P. pterocarpum* extract in different oils, surfactants, and co-surfactants.

The solubility of *P. pterocarpum* extract was investigated in different oils, surfactants, and co-surfactants to choose the system of components that can best solubilize the extract. This step is crucial to ensure the stability of the nanoemulsion to avoid drug precipitation in the parenteral dosage form. As shown in Figure 1, the highest solubility of the extract in oils was in extra virgin olive oil, followed by corn oil, sunflower oil, oleic acid, sesame oil, and isopropyl palmitate (6.61, 5.12, 4.61, 3.16, 1.62, and 0.983 mg/mL, respectively). Olive oil is a biocompatible and common carrier oil that has a high capacity to solubilize the extract [36]. Also, olive oil gives rise to a stable nanoemulsion, and it is safe for IV injection, as mentioned by Karami et al. (2019); Ren et al. (2018) [37,38]. The addition of surfactant and co-surfactant in proper ratios in the preparation of nanoemulsion is crucial to reduce the interfacial tension and to stabilize the nanoemulsion [39,40]. To ensure the safety of the nanoemulsion and its suitability for parenteral use, the proper choice of its components, as well as their quantities, is important [41]. Tween 80 is a surfactant that can best solubilize *P. pterocarpum* extract, followed by Tween 20 and finally Span 60 (7.9, 5.1, and 4.2 mg/mL, respectively). PEG 400 was chosen as a co-surfactant because it can solubilize *P. pterocarpum* extract more than glycerin (3.18 and 1.07 mg/mL, respectively). According to the solubility results, the nanoemulsion components chosen were extra virgin olive oil (oil phase), Tween 80 (surfactant), and PEG 400 (co-surfactant).

### 2.3. Construction of Pseudoternary Phase Diagram

**Figure 2:**
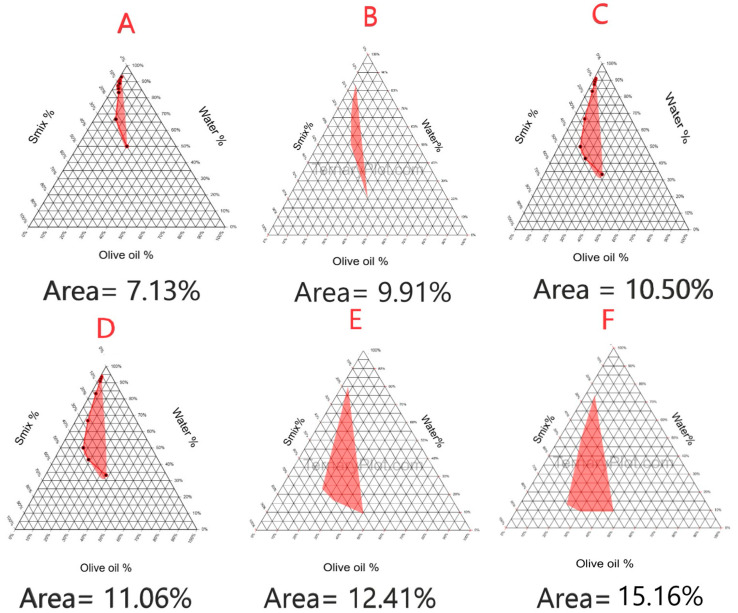
Pseudoternary phase diagram of olive oil, Smix, and water using six different Smix ratios: (**A**) (1:1), (**B**) (2:1), (**C**) (3:1), (**D**) (4:1), (**E**) (5:1), and (**F**) (6:1).

Figure 2 shows a pseudoternary phase diagram of nanoemulsion systems prepared with extra virgin olive oil, Smix (Tween 80 and PEG 400), and water. Several Smix ratios (1:1, 2:1, 3:1, 4:1, 5:1, and 6:1) were prepared. The shaded areas in each phase ternary diagram represent the clear, transparent nanoemulsion, while the unshaded parts represent the unclear, milky ones [27]. The areas of the shaded parts were 7.13%, 9.91%, 10.50%, 11.06%, 12.41%, and 15.16% for the Smix ratios 1:1, 2:1, 3:1, 4:1, 5:1, and 6:1, respectively. As reported by Khalil et al. (2015), increasing the concentration of surfactant, the isotropic region of the phase diagram gives rise to high nanoemulsion stability [42]. The Smix ratio 6:1 possesses the highest shaded area (15.16%), giving rise to a clear nanoemulsion, so it was chosen for further investigations. A further explanation is that increasing the concentration of the surfactant subsequently increases water solubilization in the nanoemulsion, giving rise to a clear system [43].

### 2.4. Characterization of the Nanoemulsion

#### 2.4.1. Percentage Transmittance, Dilution Test, Self-Emulsification Time, pH, and Viscosity

The self-emulsification time is a measure of the time needed for the nanoemulsion to emulsify with the liquid inside the body [44]. It is an important parameter in determining the efficacy and stability of the nanoemulsion. The self-emulsification time of all formulas (F1–F6) ranged from 13.11 ± 0.18 to 29.64 ± 0.66 s, as shown in Table 1. The self-emulsification time of all the formulations is good as long as it is less than 90 s for all of them, reflecting quick emulsification [44]. The pH of IV injections should be optimal to avoid local irritation [45]. The pH of formulations intended for IV injection should not exceed 9 and should not be less than 5 [46]. The pH of all the prepared formulations, F1–F6, ranged from 6.52 ± 0.11 to 7.40 ± 0.18; this indicated the suitability of the prepared nanoemulsion for IV injection [47]. Adjustment of the viscosity of the nanoemulsions intended for IV injection is crucial. Viscosity is important in determining the stability and the drug release of the nanoemulsion. Generally, on increasing the concentration of the oil, the viscosity is increased [48]. In order to obtain a stable and safe nanoemulsion intended for IV injection, the viscosity of the nanoemulsion should range from 0.54 to 0.78 cP [48]. Viscosity of the formulations ranged from 0.57 to 0.79 cP, so some of the prepared formulations may be suitable for IV injection. The typical viscosity of Tween 80 ranged from 300 to 800 cP at 25 °C, and the typical viscosity of extra virgin olive oil ranged from 80 to 100 cP at 20 °C. Regarding Smix, on increasing the concentration of the co-surfactant (PEG 400) and decreasing the surfactant’s concentration (Tween 80), the nanoemulsion’s viscosity is decreased [49]. Vice versa, the increase in Tween 80 concentration gives rise to an increase in the viscosity of the nanoemulsion, reflecting the larger droplet size of the nanoemulsion, as mentioned by Yati et al. (2017) [50].

#### 2.4.2. Determination of Drug Content, Droplet Size, Polydispersity Index (PDI), and Zeta Potential

As shown in Table 1, all formulations possess nearly 100% of the drug, which confirms the efficacy of the method of nanoemulsion preparation, which may suggest its feasibility for industrial scale [51]. In order to be suitable for IV injections, the droplet size of nanoemulsion should be less than 500 nm to avoid embolism [52]. The droplet size of all the formulations ranged from 50.12 ± 3.11 to 253.26 ± 4.11 nm, indicating their suitability for IV injection. On increasing the Smix concentration, the droplet size is decreased [53]. The Smix acts as an emulsifier that decreases the interfacial tension, which promotes emulsification, resulting in a lower droplet size [53]. On increasing the oil content, the droplet size subsequently increased [27]. This was explained by Fuhrmann et al. (2019) on the basis that after increasing the oil concentration, agglomeration of the droplets occurs, giving rise to an increase in their size [54]. It is worth mentioning that the excess amount of co-surfactant should be avoided to avoid destabilization of the nanoemulsion by micelle formation [55]. The PDI of all formulations ranged from 0.204 to 0.251; some of the prepared formulations may be homogenous and suitable for injection. PDI values up to 0.250 are considered suitable for injection, as mentioned by Séguy et al. (2020) [56]. Zeta potential is a very important factor in determining the stability of the nanoemulsion. It is a measure of the degree of repulsion between particles [57]. High zeta potential reflects high repulsive forces between particles, giving rise to high nanoemulsion stability [57]. A nanoemulsion is known to be electrostatically stable when its zeta potential is either more than +30 mV or less than −30 mV [48]. While values of zeta potential approaching zero mean attraction between particles of nanoemulsion, rapid flocculation, and reflecting low preparation stability [58]. Zeta potential of the nanoemulsion formulations ranged from −9.87 to −28.20 mV. Regarding Tween 80, despite being a non-ionic surfactant, it imparted a negative charge to the nanoemulsion. This can be explained based on the presence of fatty acid impurities or adsorption of hydroxyl ions to oil droplets [59]. Phenolic compounds in the olive oil may also be another reason for the negative charge of the nanoemulsion [60]. PEG 400 is expected to provide stability to the nanoemulsion by contributing to steric stabilization [61]. The effect of increasing the concentration of PEG 400 on the zeta potential of the nanoemulsion is non-significant. The reason for this non-significance is that the shielding effect because of PEG 400 was obvious [62].

### 2.5. TEM

**Figure 3:**
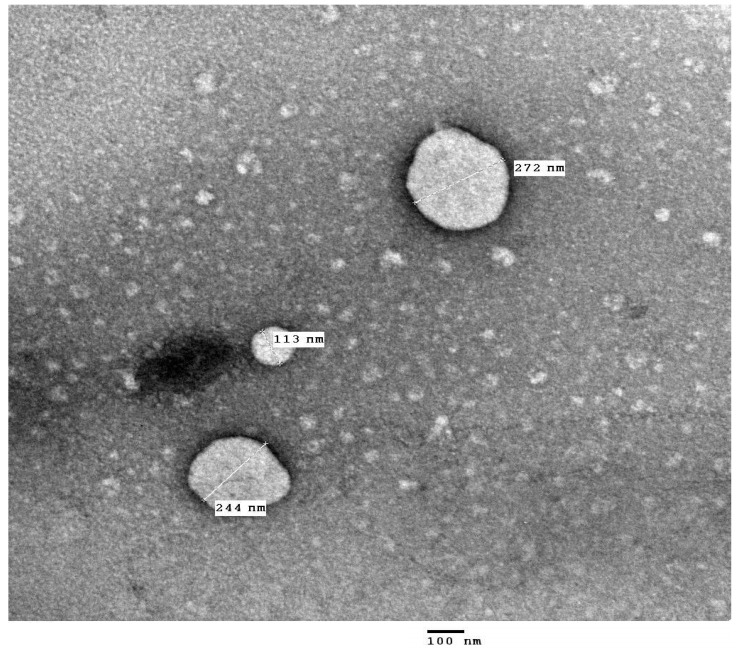
TEM micrograph of the nanoemulsion formulation “F1”.

The nanoemulsion appeared under TEM as spherical homogenous particles, as shown in Figure 3 [27,63]. This suggests that bergenin is enclosed within the spherical particles of the nanoemulsion. TEM confirms the presence of nanoemulsion in nanosize with the same size range indicated in the particle size study. The small particle size may favor absorption and bioavailability of the bergenin. The same results were mentioned by Al Zahraa and Balata (2024) and Faisal et al. (2025) [27,63].

### 2.6. In Vitro Drug Release

**Figure 4:**
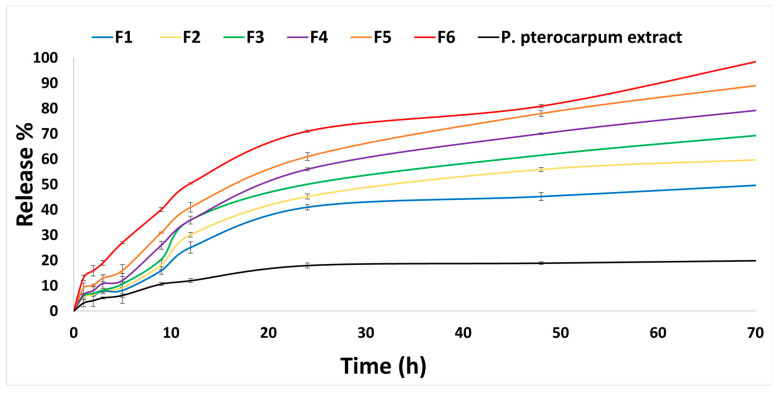
In vitro drug release of *P. pterocarpum* extract from nanoemulsions (F1–F6) in comparison with in vitro release of *P. pterocarpum* extract only in phosphate buffer (pH 7.4) at 37± 0.1 °C and 100 rpm for 72 h.

The in vitro percentage of *P. pterocarpum* extract released from the six nanoemulsion formulations is demonstrated in Figure 4, in comparison with the *P. pterocarpum* extract released. The in vitro percentage of *P. pterocarpum* extract released at pH 7.4 after 72 h was negligible (19.99%); the same results were mentioned by Bashir et al. (2022) [64]. The statistical analysis revealed a significant difference (*p* < 0.001) in comparing the percentage of *P. pterocarpum* extract released with the percentage of *P. pterocarpum* extract released from all the nanoemulsion formulations after 72 h. F6 showed the highest percentage of drug released (100%) after 72 h. The arrangement of formulations from the highest to the lowest percentage of drug released was F6 > F5 > F4 > F3 > F2 > F1. This may be attributed to the increase in Smix concentration, which is accompanied by a decrease in the droplet size and an increase in the solubility of the nanoemulsion and the percentage of the drug released, as mentioned by Wu et al. (2022) and Kotta et al. (2015) [53,65]. Being a non-ionic surfactant, Tween 80 is responsible for the increase in the contact area between the nanoemulsion and the release medium, increasing the percentage of drug released from the nanoemulsion [65]. On comparing the percentage of drug released from F6 and the other nanoemulsion formulations, a statistically significant difference was found with F1, F2, and F3 (*p* < 0.001). In addition, a statistically significant difference was revealed between F6 and F4 (*p* < 0.01). While there was a non-significant difference between F6 and F5, with *p* > 0.05. F6 was chosen for further investigation because it possesses the lowest droplet size (50.12 ± 3.11 nm), the highest zeta potential (−28.20 ± 2.90 mV), drug content (100.45 ± 0.99%), and percentage of drug released (100 ± 1.67%). In addition, F6 possesses a pH of 7.40 ± 0.18, suitable viscosity for IV injection (0.57 ± 0.15 cP), and a short emulsification time (29.64 ± 0.66 s), giving rise to quick emulsification. The in vitro release profile of the nanoemulsion formulations is extended to 72 h, which confirms gradual release over 72 h.

### 2.7. Stability Study

The stability of nanoemulsions is a very important concern, especially in those intended for IV injections [56]. The optimal formulation (F6) was kept in a refrigerator for 3 months at 4 °C. On visual inspection, the nanoemulsion was stable, homogenous, and showed no phase separation. Regarding the droplet size, zeta potential, and PDI after 3 months, they were 49.78 ± 2.11 nm, −27.6 ± 3.16 mV, and 0.298, respectively. On comparison with the initial values of droplet size, zeta potential, and PDI, which were 50.12 ± 4.11 nm, −28.2 ± 2.90 mV, and 0.279, respectively. There are nearly no changes between the initial values and after refrigeration, which confirms the stability of the nanoemulsion. Owing to the absence of coalescence, aggregation, or Ostwald ripening, which adversely affects the stability of nanoemulsion. The same drug release percentage between F6 before and after stability studies (100%) confirms the stability of the formula during storage [66]. The same results were mentioned by Séguy et al. (2020) and Bashir et al. (2022) [56,64].

### 2.8. In Vitro Hemolysis Assay

Hemolysis can be defined as the rupture of the RBCs [67]. It is the most common problem caused by the nanoperparations. Checking the hemolysis of the nanopreparations intended for IV injection is necessary [67] to ensure hemocompatibility and lack of toxicity of the preparations [56]. The hemolysis test is used to determine the degree to which the preparation destroys the RBCs [67]. The hemolytic properties of the prepared nanoformulation were analyzed quantitatively by colorimetric determination of the total hemoglobin in total human blood. This assay was initiated by Dobrovolskaia et al. (2008) [68]. The increase in hemoglobin and production of a red color is an indication of RBC destruction caused by the nanopreparation [68]. For any IV injection, if the in vitro hemolysis value is above 5%, it is considered hemolytic in vivo, and it is rejected for IV use [56]. Results of the in vitro hemolysis assay of the selected nanoemulsion formula (F6) are shown in Table S1 (Supplementary Materials). The in vitro hemolysis percentage of all the eight prepared concentrations (1000, 800, 600, 400, 200, 100, 50, and 25 μg/mL) were 1.1 ± 0.003%, 0.9 ± 0.003%, 0.7 ± 0.003%, 0.4 ± 0.002%, 0.3 ± 0.002%, 0.3 ± 0.003%, 0.0 ± 0.001%, and 0.0 ± 0.001%, respectively. All the in vitro hemolysis results of the prepared nanoemulsion concentrations were less than 5% in comparison with the control, which destroys the RBCs with an in vitro hemolysis percentage of 100 ± 0.006%. Results of the test demonstrate that the nanoemulsion is hemocompatible; in vitro and in vivo studies are needed to be done in the future. This is according to Séguy et al. (2020) [56]. Additionally, inflammation-related pathways and vascular responses play a vital role in the safety of IV therapeutics. Compounds such as SIRT6 activators have been shown to suppress LPS-induced inflammation in endothelial cells, providing insight into complementary strategies for improving IV tolerability of nanoparticle systems [69].

### 2.9. Cytotoxicity Evaluation Using Viability Assay

**Figure 5:**
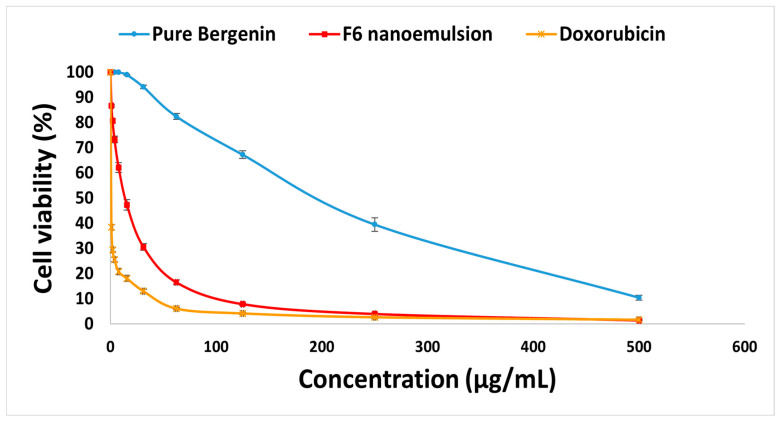
Effect of different concentrations of the selected nanoemulsion formulation (F6), and doxorubicin as a reference drug, on cell viability of hepatocellular carcinoma HepG-2 cell line. All experiments were done in triplicate and expressed as mean ± SD.

The MTT assay was used to determine the anticancer effect of bergenin on the hepatocellular carcinoma HepG-2 cell line. The antiproliferative effect of the selected nanoemulsion formulation “F6” and the reference drug doxorubicin against the HepG-2 cell line was assayed and is shown in Figure 5. Various concentrations (500, 250, 125, 62.5, 31.25, 15.6, 7.8, 3.9, 2, and 1 µg/mL) were used to detect their antiproliferative effect on the HepG-2 cell line. The antiproliferative effect of F6 on the HepG-2 cell line was significant, and it was concentration-dependent. The IC_50_ of F6 and doxorubicin were 14.19 ± 0.37 and 0.37 ± 0.05 µg/mL, respectively. While F6′s IC_50_ is higher than doxorubicin, its natural origin and hemocompatibility make it a viable candidate for further development, particularly in reducing systemic toxicity. The mechanism behind the efficacy of bergenin in the treatment of hepatocellular carcinoma may be due to its ability to decrease the release of intracellular reactive oxygen species (ROS), lower inflammatory marker expression, and decrease apoptosis [10]. The IC_50_ of pure bergenin was 60.91 ± 3.96 µg/mL against the HepG-2 cell line, as mentioned by do Nascimento et al. (2023) [70]. Results of cytotoxicity proved that F6 showed a higher anticancer effect than pure bergenin. The same results were demonstrated by Zafar et al. (2024), who proved that the nanostructured lipid carrier (NLC) containing bergenin showed more efficacy than bergenin in treatment [71]. They mentioned that the reason for this efficacy is the longer release duration of NLC compared to pure bergenin [71]. Rao et al. (2018) also confirmed that decreasing the particle size of bergenin by incorporating it into a nanosuspension improved its solubility, bioavailability, and hence its efficacy [72]. The cytotoxic effect of bergenin against the HepG-2 cell line is demonstrated by Pandey et al. (2025) [10]. Therefore, *P. pterocarpum* extract-loaded nanoemulsion (F6) is active but considerably weaker than doxorubicin. As a natural extract, *P. pterocarpum* may be preferable to synthetic chemicals due to its potentially lower toxicity [73]. These in vitro results suggest the formulation has cytotoxic activity worthy of further study as an anticancer agent.

## 3. Materials and Methods

### 3.1. Materials

*Peltophorum pterocarpum* (DC.) K. Heyne leaves (400 g) in the flowering stage were collected in October 2022 from EgyGerman Agricultural Company, Salhiya Road, Egypt. Voucher specimen PP-Leg15 was prepared and deposited at the Faculty of Pharmacy Herbarium at Zagazig University, Zagazig, Egypt. Oleic acid, corn oil, sesame oil, sunflower oil, extra virgin olive oil, and isopropyl palmitate were purchased from Botalife (Anaheim, CA, USA). Tween 80, Span 60, and glycerin were purchased from El Gomhorya Company (Zagazig, Egypt). Methanol, ethanol, de-ionized water, n-hexane, dichloromethane, ethyl acetate, potassium hydrogen phosphate, sodium chloride, potassium chloride, and sodium hydrogen phosphate were purchased from Sigma-Aldrich (Merck Ltd., Chaoyang District, Beijing, China). Dimethyl sulfoxide (DMSO), MTT, and trypan blue dye were purchased from Sigma (St. Louis, MO, USA). Fetal bovine serum, RPMI-1640, HEPES buffer solution, L-glutamine, gentamycin, and 0.25% trypsin-EDTA were purchased from Lonza (Avenue des Biolleux, 14 Parc Industriel de Petit-Rechain Verviers 4800, Belgium). All chemicals and solvents were of analytical grade.

### 3.2. Plant Extraction and Isolation of Bergenin

The leaves were washed with distilled water, air-dried, and subsequently milled, weighed (400 g), and extracted with 70% ethanol (15 L) three times. The resulting ethanolic extract was evaporated under reduced pressure to give 60 g of dry residue. A total of 40 g of the dried ethanolic extract was suspended in a methanol-water mixture (1:9, 100 mL) and partitioned against n-hexane (6 × 50 mL), dichloromethane (5 × 50 mL), and ethyl acetate (40 × 500 mL). Each fraction was washed with distilled water, dried over anhydrous sodium sulfate, and distilled off under reduced pressure at 50 °C to afford an n-hexane-soluble fraction (0.7 g), a dichloromethane-soluble fraction (1.1 g), and an ethyl acetate-soluble fraction (2.6 g). The ethyl acetate fraction was dissolved in methanol and allowed to precipitate. The precipitate was dissolved in a sufficient quantity of methanol for further crystallization to obtain pure bergenin.

### 3.3. Spectral Analysis (ES-MS, 1H-NMR, 13C-NMR, HSQC, and HMBC)

The structural elucidation of bergenin was carried out using a UV spectrophotometer (Shimadzu UV-1201, Kyoto, Japan), ES-MS, ^1^H-NMR, ^13^C-NMR, HSQC, and HMBC spectral analysis. Mass spectrometry was carried out using high-performance liquid chromatography-mass spectrometry (HPLC-PDA-ESI-MS/MS, Waters Corporation, Milford, CT, USA). The bergenin sample was subjected to HPLC analysis on ESI-MS positive and negative ion acquisition modes on a XEVO TQD triple quadrupole instrument. The sample was dissolved in HPLC-grade methanol and filtered through a 0.2 μm membrane disc filter. The resulting solution concentrations were in the range of 0.2 to 0.5 mg/mL. The reverse-phase separations were performed (ACQUITY UPLC BEH C18 Column, 1.7 μm–2.1 × 50 mm; 50 mm × 1.2 mm inner diameter; 1.7 μm particle size) at a flow rate of 0.2 m/mL. A previously reported gradient program for this analysis was demonstrated by Hassan et al. (2019) [74].

The mobile phase comprised acidified water containing 0.1% formic acid (A) and acidified methanol containing 0.1% formic acid (B). The employed elution conditions were 0–2 min, isocratic elution at 10% B; 2–5 min, linear gradient from 10 to 30%, B; 5–15 min, linear gradient from 30% to 70% B; 15–22 min, linear gradient from 70% to 90% B; and 22–25 min, isocratic elution at 90% B. Finally, washing and reconditioning of the column were done. Electrospray ionization (ESI) was performed in both negative and positive ion modes to obtain more data. The parameters for analysis were set using negative ion mode. The source temperature was 150 °C, the cone voltage was 30 eV, the capillary voltage was 3 kV, the desolvation temperature was 440 °C, the cone gas flow was 50 L/h, and the desolvation gas flow was 900 L/h. Mass spectra were detected in the ESI between *m*/*z* 100 and 1000 atomic mass units. Chemical constituents were identified by their ESI– QqQLIT–MS/MS spectra and fragmentation patterns. The peaks and spectra were processed using the MassLynx 4.1 software and tentatively identified by comparing their retention time (Rt) and mass spectrum with the reported data. ^1^H- and ^I3^C-NMR spectra were obtained using an NMR spectrometer (Bruker, Switzerland), 400 and 100 MHz, respectively. Chemical shifts were given in ppm with the tetramethylsilane (TMS) as the internal standard.

### 3.4. Standardization of P. pterocarpum Extract

The standardization of bergenin in its extract was made using HPLC to detect the amount of bergenin crystals in *P. pterocarpum* extract [75]. The pure bergenin solution was prepared by dissolving pure bergenin in methanol at a concentration of 1 mg/mL [75]. One gram of the *P. pterocarpum* extract was dissolved in 100 mL of methanol. Serial dilutions were prepared from the extract of concentrations 2.5, 10, 20, 30, 40, and 50 µg/mL. All samples were ultrasonicated (ultrasonic bath sonicator, Athena Instruments Pvt Ltd., Thane, Mumbai, India) for 30 min at 80 °C. Bergenin in its solid state is stable upon heating [27,76], then filtered using a nylon membrane filter of pore size 0.45 µm and diameter 47 mm (FILTER-LAB^®^, Barcelona, Spain). Samples were injected into HPLC; a quaternary pump and a solvent chamber with an auto-sampler injection system were both parts of the Alliance HPLC instrumentation (Waters, USA). The column used was Inertsil C18 (250 × 4.6 mm, 4.6 μm); the mobile phase was a mixture of 0.1% formic acid pH (buffer) and acetonitrile [20:80] [74]. A lab pH meter (model AD1030, Adwa Hungary Kft., Szeged, Hungary, Romania) was used to adjust the mobile phase’s pH to the desired level. The HPLC was adjusted at a temperature of 40 °C, a flow rate of 1.0 mL/min, and a wavelength of 275 nm. The data were gathered with the help of the Empower 3 program. All results were calculated in Microsoft Excel 2016 (Microsoft Corporation, USA).

### 3.5. Solubility of P. pterocarpum Extract in Different Oils, Surfactants, and Co-Surfactants

Saturation solubility of *P. pterocarpum* extract in different oils, surfactants, and co-surfactants was determined. A total of 500 mg of the extract, containing a bergenin dose (10 mg) [77], was added in sealed glass vials to 2 mL of each of different oils (extra virgin olive oil, sunflower oil, sesame oil, oleic acid, and isopropyl palmitate), different surfactants (Tween 80 and Span 60), and co-surfactants (PEG 400 and glycerin) [27]. The vials were in a thermostatically controlled shaker water bath (SW-20C, Julabo Labortechnik GmbH, Seelbach, Germany) at 25 °C, and they were shaken at 100 rpm for 24 h. A centrifuge (Centrifuge, Model Z 300 K, Hermle Labortechnik GmbH, Wehingen, Germany) was used for sample centrifugation for 30 min at 20 °C, 15,000 rpm. The resulting supernatants from all formulas were diluted using methanol and assayed using a UV spectrophotometer (Genesys 10S UV-VIS, Thermo Spectronic, Waltham, MA, USA) at 275 nm, and methanol was used as a blank.

### 3.6. Construction of Pseudoternary Phase Diagram

Oils and Smix (a surfactant and co-surfactant mixture) that can best solubilize *P. pterocarpum* extract were chosen for the construction of the pseudoternary phase diagram. The method used for the construction of the phase diagram was the aqueous titration method, as mentioned by Azeem et al. (2009) [41]. Smix was used at six different weight ratios (1:1, 2:1, 3:1, 4:1, 5:1, and 6:1). De-ionized water was added dropwise to the mixture of oil and Smix by titration, and then the mixture was vortexed using a vortex mixer (Type 16700 mixer, Thermoline Corporation, Dubuque, IA, USA) until the formation of a clear, homogenous nanoemulsion. The endpoint of titration is before the formation of a milky nanoemulsion. On the construction of all phase diagrams as shown in Figure 4, the phase diagram possessing the highest surface area was chosen for the upcoming investigations [27].

### 3.7. Preparation of P. pterocarpum Extract-Loaded Nanoemulsion

Six nanoemulsion formulations were prepared (F1–F6); their composition is shown in Table 2. In glass vials, the same dose (500 mg of *P. pterocarpum* extract) in all formulations was dissolved separately in the given amounts of oil (olive oil), surfactant (Tween 80), and co-surfactant (PEG 400) at each surfactant and co-surfactant ratio, and the 500 mg of the extract was added additionally to the weight of the oil: Smix in each nanoemulsion formulation (F1–F6). The selected oil was then incorporated with vortexing. Then the resultant mixtures were mixed using a vortex mixer, and then the de-ionized water was added to each formulation and mixed using a magnetic stirrer (Type MM5, VELP China Co. Ltd., Shanghai, China) until the formation of a clear, homogeneous nanoemulsion. The prepared formulations were kept at room temperature and are subject to the upcoming investigations.

### 3.8. Characterization of the Nanoemulsion Formulations

#### 3.8.1. Percentage Transmittance, Dilution Test, and Self-Emulsification Time

For the measurement of percentage transmittance, 1 mL of each formulation was diluted one hundred times using de-ionized water. The percentage transmittance of each formulation was measured by a UV spectrophotometer at λmax 630 nm against the blank (de-ionized water). The formula is classified as grade A if its percentage transmittance is close to 100% [78]. To detect phase separation, 10 mL of de-ionized water was added to 1 mL of each nanoemulsion formulation, and the separation was observed [79]. To determine self-emulsification time, 1 mL of each formulation was added to 500 mL of de-ionized water in a Pharma Test Dissolution Tester, rotating paddle type II (SP6-400 Hamburg, Germany) at 50 rpm at 37 °C. The time needed for the formation of a homogeneous nanoemulsion was recorded [80].

#### 3.8.2. pH and Viscosity Determination

Measurement of pH was made using a digital pH meter (ELICO LI 200, Hyderabad, India). A total of 30 mL of each formulation was prepared, and the glass electrode of the pH meter was dipped in each formulation at 25 °C [27]. The pH value of each formulation was measured three times. Viscosities of the formulations were measured using a viscometer (Visco Star-R, FUNGILAB S.A., San Feliú de Llobregat, Barcelona, Spain).

#### 3.8.3. Determination of Droplet Size, PDI, and Zeta Potential

The prepared droplets of nanoemulsion were analyzed for their particle size and size distribution in terms of the average volume diameters, PDI, and zeta potential by photon correlation spectroscopy using particle size analyzer Dynamic Light Scattering (DLS) (Zetasizer Nano ZN, Malvern Panalytical Ltd., Malvern, UK) at 25 °C and a fixed angle of 173° using disposable zeta cells. Samples were analyzed in triplicate. Determination of the zeta potential was performed using the same equipment [27,81].

#### 3.8.4. Drug Content Measurement

The nanoemulsion (2 mL) containing 500 mg of *P. pterocarpum* extract was diluted using methanol. The drug content of the nanoemulsion was determined by a UV spectrophotometer at λmax 275 nm; methanol was used as a blank. All measurements were done three times, and standard deviations were calculated. Drug content was calculated using the following equation (Equation (1)) [27,82]:

(1) $$Drug\;content=\frac{Analyzed\;drug\;content}{Theoretical\;drug\;content}\times 100$$

#### 3.8.5. Transmission Electron Microscopy (TEM)

TEM imaging is a way to confirm the shape and size of the prepared nanoemulsion. One drop of the selected nanoemulsion formulation was added to carbon-coated copper grids (CCG), stained for 4 min using uranyl acetate solution (2% *w*/*v*), and then the grid was kept at room temperature until evaporation of water [83,84]. Transmission electron microscope (JEOL JEM-1010, JEOL Ltd., Akishima, Tokyo, Japan) at the regional center for mycology and biotechnology (RCMB), Al-Azhar University, Cairo, Egypt.

### 3.9. In Vitro Drug Release

In vitro drug release of the nanoemulsion and *P. pterocarpum* extract was carried out using a release medium of phosphate buffer pH 7.4, prepared using de-ionized water and 1% Tween 80 as a surfactant. The freshly prepared release medium (20 mL) was added to each glass beaker containing 2 mL of the nanoemulsion formulations inserted into cellophane dialysis bags (molecular weight cut-off 12,000–14,000 Da), which were soaked in the buffer a whole night before the release day. The glass beakers were placed in a thermostatic shaker water bath at 100 rpm, 37 ± 0.1 °C. Samples of 2 mL were withdrawn at a time interval of 0.5, 1, 2, 3, 4, 8, and 24 h [27]. The volume was replaced with an equal volume (2 mL) of freshly prepared phosphate buffer for the sink conditions to be maintained. A UV spectrophotometer was used for analysis of all the withdrawn samples at λmax 275 nm against a freshly prepared phosphate buffer as a blank. All experiments were done three times, and then the overall percentage of drug released was calculated. A curve of the cumulative percentage of drug released by all the formulas and *P. pterocarpum* extract against time was plotted. The formulas were compared with each other to choose the optimum formula possessing the highest percentage of drug released.

### 3.10. Stability Study

The selected nanoemulsion formulation was kept in the refrigerator for three successive months at 4 °C. The stability of the nanoemulsion was evaluated by measuring its particle size, PDI, zeta potential, in vitro percentage of drug released, and visual inspection of its clarity [27].

### 3.11. In Vitro Hemolysis Assay

Using the Bulmus et al. (2003) method, the in vitro hemolytic assay was carried out [85], using human red blood cells that were freshly collected. RBCs were then subjected to a triplicate wash with 150 mM NaCl (2500 rpm, 10 min). The plasma cells were removed, and they were suspended in phosphate-buffered saline (PBS, pH 7.4) to reach a concentration of 2% RBCs. Double-folded dilutions of concentrations (1000, 800, 600, 400, 200, 100, 50, and 25 μg/mL) of the nanoemulsion were mixed with 2% RBCs solutions. The final reaction mixture volume was made up to 1 mL by adding phosphate saline. Then the mixture was kept for 1 h in a water bath at 37 °C. The mixture was then centrifuged for 15 min at 2500 rpm. The supernatant was used to measure the optical density at λmax 546 nm, using phosphate buffer saline as a blank. The positive control in this experiment was de-ionized water. All measurements were done in triplicate, and then the mean ± S.D. was calculated using the following equation (Equation (2)):

(2) $$Percentage\;hemolysis=\frac{\left(Absorbance\;of\;sample-absorbence\;of\;blank\right)}{Absorbence\;of\;blank}\times 100$$

The cells were grown on RPMI-1640 medium supplemented with 10% inactivated fetal calf serum and 50 µg/mL gentamycin. The cells were maintained at 37 °C in a humidified atmosphere with 5% CO_2_ and were subcultured two to three times a week.

### 3.12. Cytotoxicity Evaluation Using Viability Assay

The MTT assay was carried out using the human hepatocellular cancer cell line (HepG-2 cell line). The tumor cell lines were supplied from the American Type Culture Collection (ATCC, Rockville, MD, USA). Using Corning^®^ medium at a concentration of 5 × 10^4^ cells/well, cell lines were suspended in 96-well tissue culture plates. Cell lines were incubated for 24 h. F6 nanoemulsion was compared with doxorubicin as a reference; each was added separately to the 96-well plates in three replicates. Each sample should have 12 concentrations (500, 250, 125, 62.5, 31.25, 15.6, 7.8, 3.9, 2, 1, and 0 µg/mL); the concentrations of the sample and doxorubicin were fixed to compare the cytotoxicity of both of them. For each well plate, six vehicle controls with 0.5% DMSO were used; this concentration is safe and non-toxic [86]. Incubation took place for 48 h, and then the MTT test was taken to determine the number of viable cells. Replacement of the media in the well plates was executed with 100 µL of fresh culture RPMI 1640 medium. Then 5 mg of MTT in 1 mL of PBS was added to each well (both treated and untreated ones). A total of 5% CO_2_ was added to all the plates at 37 °C for 4 h. A volume of 85 µL of the media was discarded, then in each well 50 µL of DMSO was inserted and incubated for 10 min at 37 °C. Using a microplate reader (SunRise, TECAN, Inc., Chapel Hill, NC, USA), the optical density (OD) was determined at λmax 590 nm. Using the following equation (Equation (3)), the number of viable cells and the viability percentage were calculated.

(3) $$Viability\;percentage=\left[\frac{ODT}{ODC}\right]\times 100$$

where ODT is the mean OD of the treated cells, and ODC is the mean OD of the untreated cells. To obtain the survival curve, the concentration of the drug was plotted against the number of surviving cells. The concentration needed to inhibit 50% of the cells (IC_50_) was calculated with the aid of the plot [87,88].

### 3.13. Statistical Analysis

Statistical analysis for the results was carried out using two-way ANOVA applied by GraphPad Prism 5, CA, Graphpad by dotmatics, Boston, MA, USA. The results are said to be significant if the *p*-value is <0.001.

## 4. Conclusions

The nanoemulsion of *P. pterocarpum* extract was prepared using extra virgin olive oil, Tween 80 as a surfactant, and PEG 400 as a co-surfactant. After construction of the pseudoternary phase diagram, the nanoemulsion prepared using a 6:1 Smix ratio was chosen, as it possessed the highest surface area, 15.16%. Six nanoemulsion formulations were prepared by increasing the oil: Smix ratio. F6 was chosen for further investigations as it is transparent and clear. It also possesses the lowest droplet size (50.12 ± 3.11 nm), the highest zeta potential (−28.20 ± 2.90 mV), drug content (100.45 ± 0.99%), and percentage of drug released (100 ± 1.67%). In addition, F6 possesses a pH of 7.40 ± 0.18, a suitable viscosity for IV injection (0.57 ± 0.15 cP), and a short emulsification time (29.64 ± 0.66 s), giving rise to quick emulsification. F6 showed shelf-life stability, possessing nearly the same particle size, zeta potential, and in vitro drug release after 3 months. Results of the hemocompatibility study revealed that F6 is hemocompatible, indicating potential suitability for IV administration. It shows anticancer activity against the HepG-2 cell line (IC_50_ = 14.19 ± 0.37 µg/mL). *P. pterocarpum* extract-loaded nanoemulsion may be used for the treatment of hepatocellular carcinoma, possessing efficacy and implying presumed safety. Our future studies will include in vivo efficacy and toxicity assays to further support the clinical potential of this nanoemulsion.

## Figures and Tables

**Table 1:** Characterization of the nanoemulsion formulations (F1–F6) in terms of drug content, droplet size, zeta potential, PDI, percentage transmittance, dilution, self-emulsification time, pH, and viscosity.

Formula	Drug Content (%)	Droplet Size (nm)	Zeta Potential (mV)	PDI	Percentage Transmittance (%)	Dilution	Self-Emulsification Time (s)	pH	Viscosity (cP)
F1	99.89 ± 0.06	253.26 ± 4.11	−9.87 ± 0.45	0.225	100.03 ± 0.01	√	13.11 ± 0.18	6.52 ± 0.11	0.79 ± 0.97
F2	100.01 ± 0.01	145.36 ± 3.98	−12.83 ± 1.45	0.209	99.99 ± 0.15	√	15.23 ± 0.55	6.80 ± 0.17	0.73 ± 0.77
F3	100.06 ± 0.11	121.65 ± 2.17	−14.03 ± 3.99	0.204	100.01 ± 0.18	√	17.88 ± 2.00	7.11 ± 0.77	0.69 ± 0.58
F4	100.11 ± 0.05	89.50 ± 1.19	−18.46 ± 5.71	0.219	99.89 ± 0.21	√	21.55 ± 0.11	7.12 ± 0.81	0.61 ± 0.26
F5	100.09 ± 0.11	70.11 ± 3.10	−21.80 ± 1.11	0.251	100.00 ± 0.19	√	25.11 ± 0.17	6.86 ± 0.32	0.59 ± 0.22
F6	100.45 ± 0.99	50.12 ± 3.11	−28.20 ± 2.90	0.229	100.01 ± 0.11	√	29.64 ± 0.66	7.40 ± 0.18	0.57 ± 0.15

‘√’ means ‘no separation’.

**Table 2:** Composition of the nanoemulsion formulations selected for loading of the extract.

Formula	Oil:Smix Ratio	Olive Oil (g)	Smix (g)	De-Ionized Water (g)
F1	1:1	0.500	0.500	0.100
F2	1:2	0.330	0.660	0.100
F3	1:3	0.250	0.750	0.150
F4	1:4	0.200	0.800	1.000
F5	1:5	0.166	0.830	2.800
F6	1:6	0.143	0.858	3.000

## Data Availability

The original contributions presented in this study are included in the article/Supplementary Material. Further inquiries can be directed to the corresponding authors.

## Supplementary Materials

The following supporting information can be downloaded at: https://www.mdpi.com/article/10.3390/ph18121818/s1, Figure S1. A: IUPAC Numbering of Bergenin, B: Important COSY, and C: HMBC- Correlations. Figure S2. HPLC chromatogram of pure bergenin at λmax 275 nm. Figure S3. Peltophorum pterocarpum plant a) the entire plant, b) leaves. Table S1. Results of the RBCs hemolysis test for the selected nanoemulsion formulation (F6).

## Abbreviations

The following abbreviations are used in this manuscript:

HCC	Hepatocellular carcinoma
Smix	Surfactant to co-surfactant mixture
*P. pterocarpum*	*Peltophorum pterocarpum*
RFA	radiofrequency
MWA	microwave
TACE	trans-arterial chemoembolization
TARE	trans-arterial radioembolization
EASL	European Association for the Study of the Liver
AASLD	American Association for the Study of Liver Diseases
BCLC	Barcelona Clinical Liver Cancer
PDI	poly dispersibility index
DLC	Dynamic Light Scattering
TEM	Transmission electron microscopy
HepG-2 cell line	human hepatocellular cancer cell line
OD	optical density
IC_50_	The concentration needed to inhibit 50% of the cells
