# Supplementary material for: Unveiling the Cytotoxicity Potential of Nanoemulsion of Peltophorum pterocarpum Extract: A Natural Hemocompatible Injection Competing with Doxorubicin
Source: Pharmaceuticals (Basel). 2025 Nov 28;18(12):1818. doi: 10.3390/ph18121818 (PMC12735563; doi:10.3390/ph18121818)
Supplement: Supplementary file 1 [file pharmaceuticals-18-01818-s001.zip › pharmaceuticals-3953785-supplementary.pdf]

Supplementary data

Characterization of bergenin

The structural elucidation of the compound was carried out using UV, ES-MS, ^{1}H-NMR, ^{13}C-NMR, HSQC, and HMBC spectral analysis. Bergenin (Figure S1): white crystals with Rf value 0.45 (using solvent system dichloromethane: methanol (8.5:1.5)). UV: λmax (MeOH) nm: 275. ^{1}H-NMR (400 MHz, CD_3OD, δ_H ppm): 7.07 (^{1}H, s, H-7), 4.95 (1H, d, J = 10.8 Hz, H-10b), 4.04 (2H, m, H-4a, H-4), 3.89(3H, s, H-12), 3.80 (1H, t, J = 8.4 Hz, H-2), 3.68 (2H, m, H-11), and 3.43(1H, m, H-3). ^{13}C-NMR (100 MHz, CD_3OD, δC ppm): 165.76 (C-6), 152.31 (C-8), 149.41 (C-10), 142.25 (C-9), 119.4 (C-6a), 117.27 (C-10a), 111.04 (C-7), 83.01 (C-2), 81.38 (C-4a), 75.59 (C-4), 74.22 (C-10b), 71.86 (C-3), 62.63 (C-11), and 60.89 (C-12(-OCH3)). HMBC (400 MHz, CD3OD): H-7 →C-6, C-8, C-9 and C-10a; H-12→C-9; H-10b→C-10, C-10a, C-6a and C-4a; H-3→ C11, C-4 and C-2; H-11→ C-3, C-2; H-2→ C-3; H-4→ C-3, C-10b; H-4a→ C-3, C-10a. ES-MS (-ve) m/z 327 [M-H]- (calculated for C14H15O9).

The ^{1}H NMR spectrum of the compound (Figure S1) showed signals for aromatic proton at δ_H 7.07 (1H, s, H-7), methoxy group at δ_H 3.89(3H, s, H-12) and the remaining signals at δ_H 4.95 (1H, d, J = 10.8 Hz, H-10b), 4.04 (2H, m, H-4a, H-4), 3.89(3H, s, H-12), 3.80 (1H, t, *J* = 8.4 Hz, H-2), 3.68 (2H, m, H-11), and 3.43(1H, m, H-3) for sugar moiety. In addition, the ^{13}C-NMR spectrum (Figure S2) showed fourteen signals one for carbonyl group, one signal for methoxy group, six signals for aromatic ring and six signals for sugar moiety. The protons and carbons assignment were confirmed by the aid of HMBC and HSQC spectra (Figure S3 and Figure S4). Where the HMBC spectrum (Figure S3) showed that the aromatic proton signal at δH 7.07(H-7) correlated to a carbonyl carbon at δC 165.76 (C-6) and two oxygenated aromatic carbons at δ_C 142.25 (C-9) and 152.31 (C-8). The aromatic proton (H-7) also correlated to aromatic carbon at δ_C 117.27 (C-10a). Therefore, a carbonyl group could be attached at C-6a that is ortho to the carbon bearing the only aromatic proton. Furthermore, the methoxy proton signal at δH 3.89 correlated to an aromatic carbon at δ_C 142.25 (C-9), thus implying that, the methoxy group could be attached on the aromatic ring at C-9 which was meta to C-7 but para to C-6a- bearing the benzoyl carbon (C-6).

It was also noted from HMBC spectrum that H 10b, at 4.95 ppm (anomeric proton) correlated to one oxygenated aromatic carbon at δC 149.41 (C-10), two other aromatic carbons at δC 117.27 (C-10a) and δC 119.4 (C-6a) and aliphatic carbon at δC 81.83(C-4a); the proton signal at δH 3.43(H-3) correlated to carbons at δC 62.63 (C-11), δc 75.59 (C-4) and δC 83.01 (C-2); the proton signal at δH 3.68 (H-11) correlated to carbons at δC 71.86 (C-3) and 83.01 (C-2); the proton signal at δH 3.82 (H-2) correlated to carbon at δC 71.86 (C-3); the proton signal at δH 4.04 (H-4 and H-4a) correlated to carbons at δC 71.86 (C-3), 74.22(C-10b) and 117.27(C-10a). The correlation of each proton with its corresponding carbon was evidenced from the HSQC spectrum which showed correlations between C and H atoms: 3.80, 83.01 (CH aliphatic, C-2); 3.43, 71.86 (CH aliphatic, C-3); 4.04, 75.59 (CH, aliphatic, C-4); 4.04, 81.38 (CH aliphatic, C-4a); 7.07, 111.04 (CH aromatic, C-7); 4.95, 74.22 (CH aliphatic, C-10b); 3.68 ,62.63 (CH_2 aliphatic, C-11) and 3.89, 60.89 (-OCH3). HSQC correlations confirmed that only one proton (δH 6.99, H-7) was attached to the aromatic carbon (δC 110.2, C-7). The compound was confirmed to be bergenin by careful inspection of 1D and 2D NMR spectroscopic analysis and when compared with the data reported by Zhang et al., 2023, Agber et al., 2020, and Qin et al., 2009 respectively [34-36].

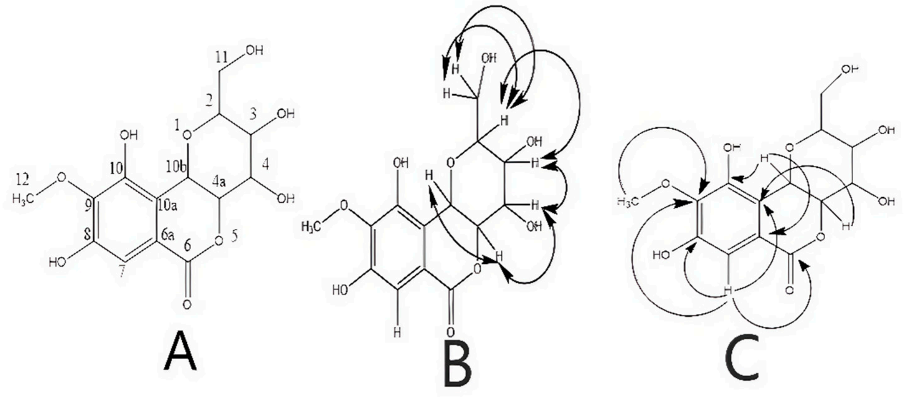

Figure S1. A: IUPAC Numbering of Bergenin, B: Important COSY, and C: HMBC- Correlations.

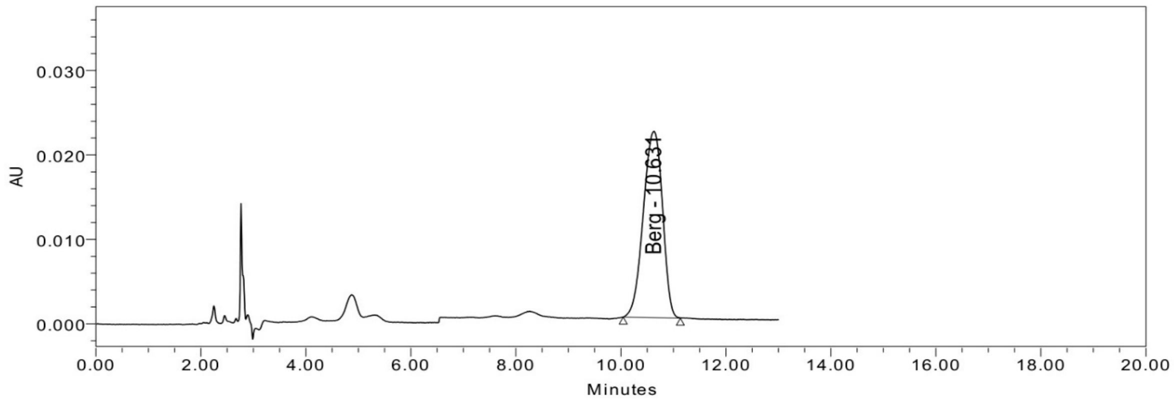

Figure S2. HPLC chromatogram of pure bergenin at λmax 275 nm.

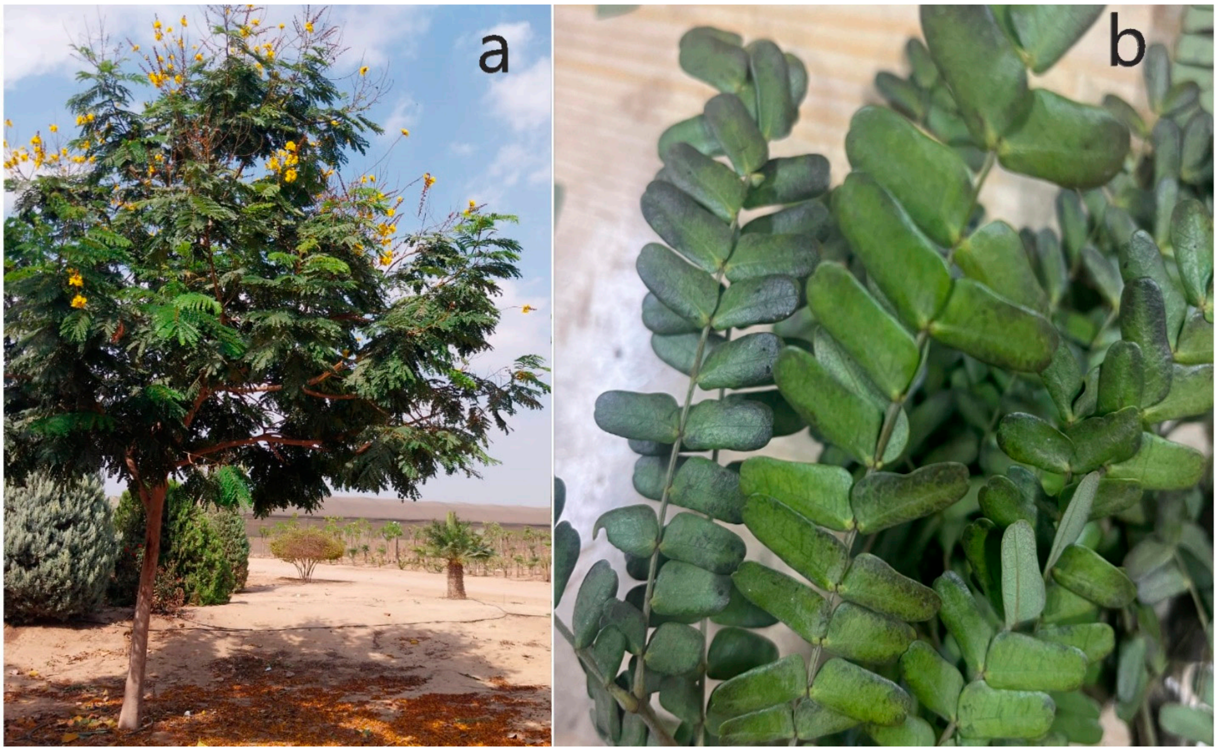

Figure S3. *Peltophorum pterocarpum* plant a) the entire plant, b) leaves.

Table S1. Results of the RBCs hemolysis test for the selected nanoemulsion formulation (F6).

Sample Conc. (ug/mL).	OD R1	OD R2	OD R3	Absorbance mean of sample with RBCs	Absorbance mean of sample with isotonic solution	Hemolysis %	SD	SE
Control Complete Hemolysis	1.22	1.231	1.226	1.226		100.0	0.006	0.002
1000	0.061	0.055	0.058	0.058	0.045	1.1	0.003	0.001
800	0.044	0.048	0.05	0.047	0.036	0.9	0.003	0.001
600	0.035	0.036	0.031	0.034	0.025	0.7	0.003	0.001
400	0.024	0.021	0.022	0.022	0.018	0.4	0.002	0.000
200	0.016	0.019	0.015	0.017	0.013	0.3	0.002	0.001
100	0.013	0.011	0.008	0.011	0.007	0.3	0.003	0.001
50	0.006	0.005	0.007	0.006	0.006	0.0	0.001	0.000
25	0.003	0.003	0.001	0.002	0.002	0.0	0.001	0.000

Where OD is the optical density, R1 is the first run, R2 is the second run, R3 is the third run, SD is the standard deviation, and SE is the standard error.
